# Supplementary material for: Time‑resolved multi-omic analysis of paclitaxel exposure in human iPSC‑derived sensory neurons unveils mechanisms of chemotherapy‑induced peripheral neuropathy
Source: Cell Death Dis. 2026 Feb 10;17(1):211. doi: 10.1038/s41419-026-08445-2 (PMC12921266; doi:10.1038/s41419-026-08445-2)
Supplement: Supplementary file 15 — Dataset 1. [file 41419_2026_8445_MOESM15_ESM.zip › CDD_Open_Data/04_RNA_Seq/Timeline_panel_plots_etc_2023-01-23_1609.zip!/Panel_Plots_etc/Evidence_Plots/72h/evidence_plot_standard contrasts_tmod_72h_PTX_vs_DMSO_ID0_LI.M1.0_integrin cell surface interactions (I).pdf]

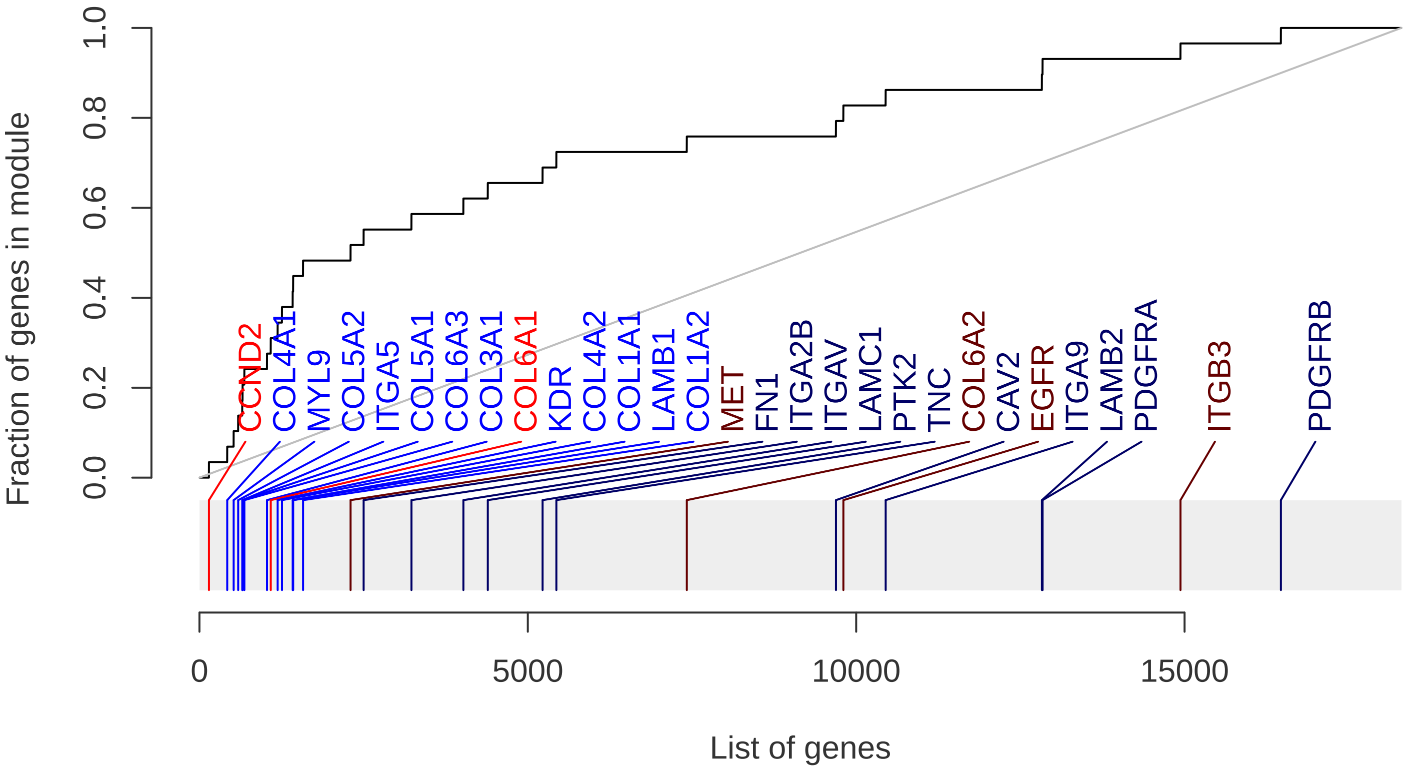

Fraction of genes in module
0.0
0.2
0.4
0.6
0.8
1.0
CCND2
COL4A1
MYL9
COL5A2
ITGA5
COL5A1
COL6A3
COL3A1
COL6A1
KDR
COL4A2
COL1A1
LAMB1
COL1A2
MET
FN1
ITGA2B
ITGAV
LAMC1
PTK2
TNC
COL6A2
CAV2
EGFR
ITGA9
LAMB2
PDGFRA
ITGB3
PDGFRB
0
5000
10000
15000
List of genes
